# Integrated Workflow for Drug Repurposing in Glioblastoma: Computational Prediction and Preclinical Validation of Therapeutic Candidates

**DOI:** 10.3390/brainsci15060637

**Published:** 2025-06-13

**Authors:** Nazareno Gonzalez, Melanie Pérez Küper, Matías Garcia Fallit, Jorge A. Peña Agudelo, Alejandro Nicola Candia, Maicol Suarez Velandia, Ana Clara Romero, Guillermo Videla Richardson, Marianela Candolfi

**Affiliations:** 1 Instituto de Investigaciones Biomédicas (INBIOMED, CONICET-UBA), Facultad de Medicina, Universidad de Buenos Aires, Buenos Aires C1121ABG, Argentina; gonzalez.nazareno1@gmail.com (N.G.); melaniepk@hotmail.com (M.P.K.); mati.garciafallit@gmail.com (M.G.F.); jarmando2192@gmail.com (J.A.P.A.); alenicola90@gmail.com (A.N.C.); mmauriciosuarez@unicolmayor.edu.co (M.S.V.); aromero@fmed.uba.ar (A.C.R.); 2 Departamento de Fisiología, Biología Molecular y Celular, Facultad de Ciencias Exactas y Naturales, Universidad de Buenos Aires, Buenos Aires C1428AQK, Argentina; 3 Fundación Para la Lucha Contra las Enfermedades Neurológicas de la Infancia (FLENI), Buenos Aires C1121A6B, Argentina; willyvidelar@hotmail.com

**Keywords:** glioblastoma, drug repurposing, blood-brain barrier, Daporinad, predictive modeling, NAMPT inhibitor, personalized medicine, combination therapy

## Abstract

Background: Glioblastoma (GBM) remains a significant challenge in oncology due to its resistance to standard treatments including temozolomide. This study aimed to develop and validate an integrated model for predicting GBM sensitivity to alternative chemotherapeutics and identifying new drugs and combinations with therapeutic potential. Research Design and Methods: We analyzed drug sensitivity data for 272 compounds from CancerRxTissue and employed in silico algorithms to assess blood-brain barrier permeability. The model was used to predict GBM sensitivity to various drugs, which was then validated using GBM cellular models. Alternative drugs targeting overexpressed and negative prognostic biomarkers in GBM were experimentally validated. Results: The model predicted that GBM is more sensitive to Etoposide and Cisplatin compared to Temozolomide, which was confirmed by experimental validation in GBM cells. We also identified novel drugs with high predicted sensitivity in GBM. Daporinad, a NAMPT inhibitor that permeates the blood-brain barrier was selected for further preclinical evaluation. This evaluation supported the in silico predictions of high potential efficacy and safety in GBM. Conclusions: Our findings using different cellular models suggest that this computational prediction model could constitute a valuable tool for drug repurposing in GBM and potentially in other tumors, which could accelerate the development of more effective cancer treatments.

## 1. Introduction

Glioblastoma (GBM) is the most common and aggressive primary malignant brain tumor in adults, with a median survival of only 15–18 months despite advances in surgery, radiotherapy, and chemotherapy with temozolomide (TMZ) [1,2,3,4,5]. The 2021 WHO classification of central nervous system tumors refined GBM diagnostics by incorporating molecular features, such as IDH mutation status [2,6], but this has not yet translated into improved treatment outcomes [7,8,9,10,11]. With therapeutic progress stagnating and survival rates remaining dismal, there is an urgent need for novel, more effective, and readily translatable strategies. In this context, drug repurposing has emerged as a promising approach to accelerate therapeutic discovery using approved or clinically tested compounds. 

There is a critical need to explore new therapies for GBM. Advances in cancer genomics, driven by initiatives like the Human Genome Project [12], have enabled researchers to identify biomarkers that improve cancer diagnosis, prognosis, and treatment. Large datasets such as those from the Gene Expression Omnibus (GEO) [13] and The Cancer Genome Atlas (TCGA) have proven invaluable for these discoveries [14,15]. Furthermore, molecular characteristics such as gene mutations and expression profiles play a key role in predicting drug response, making bioinformatic approaches essential for identifying new therapies [16,17]. In particular, Li et al. [18] developed a computational model that predicts drug sensitivity values across different cancer types using gene expression data, enabling the in silico prediction of effective therapies [19,20]. Building on this approach, we designed a novel GBM-specific workflow that integrates drug response prediction with key pharmacological and biological filters—including blood-brain barrier (BBB) permeability, differential expression of drug targets between tumor and normal brain tissue, and prognostic relevance of those targets. Importantly, we complemented our computational pipeline with experimental validation in multiple GBM models, including patient-derived cells, enhancing the biological relevance and translational potential of the drug repurposing process. This integrated strategy aims to improve the prioritization of effective therapeutic candidates specifically tailored to the unique challenges of GBM.

## 2. Materials and Methods

### 2.1. Glioma Biopsies Datasets

Clinical, genomic, and transcriptomic data from glioma patients in the TCGA LGG-GBM cohort and normal brain tissue samples from the GTEx dataset were used. Patients were stratified according to their IDH1/2 mutational status into mIDH and wtIDH groups. Expression data of DNA topoisomerase II alpha (TOP2A), Nicotinamide phosphoribosyltransferase (NAMPT), epithelial–mesenchymal markers, and canonical targetable signaling pathways were downloaded from https://tcga-data.nci.nih.gov/ accessed on 1 March 2025 via the Xena Browser developed by the University of California, Santa Cruz (UCSC) [21].

### 2.2. Drug Prediction Workflow

We used clinical and transcriptomic data from The Cancer Genome Atlas (TCGA) to identify candidate drugs for glioblastoma (GBM). Predicted ln(IC50) values for 272 drugs in TCGA glioma patients were obtained from CancerRxTissue [18]. For each drug, we first calculated the median predicted IC50 across all patients to estimate its efficacy. Then, we calculated the median of these drug-level medians to define a reference threshold for drug efficacy in GBM.

We applied a multi-criterion in silico filtering strategy to prioritize compounds. First, blood-brain barrier (BBB) permeability was predicted using two independent computational tools, cBioligand [22] and ADMETlab3.0 [23]. Next, drug target genes were evaluated for upregulation in GBM compared to normal brain tissue using publicly available transcriptomic datasets (GTEx). We also assessed the clinical relevance of each drug target by analyzing its association with patient progression-free interval (PFI) and overall survival (OS) using Kaplan–Meiers curves.

Candidate drugs were selected based on the following criteria: (1) significant upregulation of the drug target in tumor versus normal tissue; (2) predicted BBB permeability (BBB+); (3) higher predicted efficacy (lower ln(IC50)) compared to temozolomide (TMZ); and (4) negative prognostic significance of the target gene in PFI and OS.

For in vitro validation, we selected cell lines based on drug target expression profiles obtained from The Human Protein Atlas [24], aiming to include lines with variable expression levels for the targets of interest. The selected drugs were then tested in these cell lines to validate the in silico predictions.

### 2.3. Potential Combination Analyses of Temozolomide with Other Drugs

To identify potential combination therapies for GBM, we investigated the relationship between predicted TMZ efficacy and the expression of nicotinamide phosphoribosyltransferase (NAMPT), the molecular target of Daporinad. Predicted ln(IC50) values for TMZ were obtained from CancerRxTissue [18], and NAMPT mRNA expression levels were extracted from TCGA GBM transcriptomic data. Spearman correlation analysis was used to assess the association between NAMPT expression and TMZ predicted efficacy across patient samples. To further evaluate the impact of NAMPT expression, samples were stratified into “Low” and “High” groups using the median NAMPT expression as a cut-off. Differences in predicted TMZ sensitivity between these groups were compared to determine whether elevated NAMPT expression was associated with reduced TMZ efficacy, supporting the rationale for evaluating Daporinad and TMZ as a potential combination strategy.

### 2.4. Drugs

Daporinad was dissolved in dimethyl sulfoxide (DMSO) to a final concentration of 10 mM. Aliquots of the stock solution were prepared to prevent repeated freeze–thaw cycles and stored at −80 °C.

Cell culture media, including Dulbecco’s Modified Eagle Medium (DMEM; Cat# 12100046), Dulbecco’s Modified Eagle Medium: F-12 Nutrient Mix (DMEM/F-12; Cat# 12500062), Neurobasal Medium (Cat# 21103049), B-27 and N-2 supplements (Cat# A35828-01 and Cat# 17502-048, respectively), Geltrex LDEV-Free Reduced Growth Factor Basement Membrane Matrix (Cat# A14132-02), penicillin–streptomycin (Cat# 15140122), and trypsin–EDTA (0.025%, Cat# 25200114) were obtained from Gibco (Invitrogen, Carlsbad, CA, USA). Fetal bovine serum (FBS) was acquired from Natocor (Cordoba, Argentina).

### 2.5. Cell Culture

Human GBM commercial cell lines (U-251, LN-229, and U-87), and neurospheres from murine wtIDH and mIDH gliomas [25] were kindly donated by Dr. Maria G. Castro (University of Michigan, Ann Arbor, MI, USA).

Human GBM cell lines were cultured in DMEM supplemented with 5% FBS and 1% penicillin–streptomycin, pH 7.4, under 5% CO_2_ at 37 °C. Cells were dissociated with 0.05% trypsin–EDTA and subcultured every three days.

Murine GBM neurospheres were derived from wtIDH and mIDH gliomas developed by genetic engineering in the mouse brain [25], and kindly donated by Dr. Maria G. Castro (University of Michigan, Ann Arbor, MI, USA). Neurospheres were maintained in DMEM/F12 medium supplemented with 1% penicillin–streptomycin, 1X B-27, 1X N-2, 100 μg/mL Normocin, 20 ng/mL bFGF, and 20 ng/mL EGF under non-adherent conditions. Prior to experimental use, neurospheres were enzymatically dissociated into single cells using Accutase.

Patient-derived GBM cell cultures (G02, G03, G08, and G09) used in this study were previously isolated from human biopsies following relevant guidelines and national regulations [26]. The use of these cultures for biomedical research was approved by the Research Ethics Committee “Comité de Ética en Investigaciones Biomédicas de la Fundación para la Lucha contra Enfermedades Neurológicas de la Infancia (FLENI)”. These cells were cultured on Geltrex-coated Petri dishes with serum-free neurobasal medium supplemented with glucose, sodium pyruvate, PBS-BSA (7.5 mg/mL), 1X B27, 1X N2, 20 ng/mL bFGF and EGF, 2 mM L-glutamine, 2 mM non-essential amino acids, and 50 U/mL penicillin–streptomycin. Cells were harvested using Accutase.

### 2.6. In Vitro Validation of Therapeutic Potential

The antitumoral effects of chemotherapeutic and alternative drugs (Temozolomide, Cisplatin, Etoposide, and Daporinad) were assessed using a 3-(4,5-dimethylthiazol-2-yl)-2,5-diphenyltetrazolium bromide (MTT) colorimetric assay. A total of 5000 cells were seeded per well in 96-well plates. After 24 h, cells were washed and incubated with 100 μL of the respective treatments. After 72 h, the treatment was removed, and the wells were washed. Subsequently, 110 μL of MTT 450 μg/mL (Molecular Probes, Invitrogen, Thermo Fisher Scientific, Waltham, MA, USA) in Krebs–Henseleit solution was added to each well. Plates were then incubated for 4 h at 37 °C, and absorbance at 595 nm was measured. IC50 values were calculated through non-linear regression using GraphPad Prism. Each condition was tested in six technical replicates, and the experiment was independently repeated at least three times.

### 2.7. Wound Closure Assay

To assess the migratory capacity of GBM cells (U-251 and LN-229), 100,000 cells were seeded per well in 24-well plates and cultured under standard conditions until reaching 90–100% confluence. After 24 h of treatment with or without Etoposide (1 µM) or Daporinad (10 nM), a linear wound was created in the monolayer using a sterile 200 µL pipette tip. Detached cells were removed by washing twice with phosphate-buffered saline (PBS), and fresh medium (with or without treatment) was added. Phase-contrast images of the same wound area were taken immediately after scratching (time 0) and at 24 and 48 h using a phase-contrast microscope. Wound area was measured using ImageJ software 1.x, and migration rate was calculated as the percentage of wound closure relative to time 0. Each condition was performed in triplicate, and the experiment was independently repeated at least three times.

### 2.8. Statistical Analyses

Statistical analyses were performed using GraphPad Prism version 8 software. Data normality was assessed with the Kolmogorov–Smirnov test prior to conducting parametric statistical tests. Continuous variables were compared using Student’s *t*-test or one-way analysis of variance (ANOVA). Correlations were evaluated using Spearman correlation analysis. Kaplan–Meier curves were analyzed using the Log-rank test. Differences were considered significant when the *p*-value < 0.05. All quantitative experiments were performed in biological triplicate with technical duplicates. Data are presented as mean ± standard deviation, and appropriate statistical tests were applied as described below.

## 3. Results

### 3.1. Efficacy Prediction Model for Chemotherapeutic Drugs with Potential Effect in GBM

**Figure 1:**
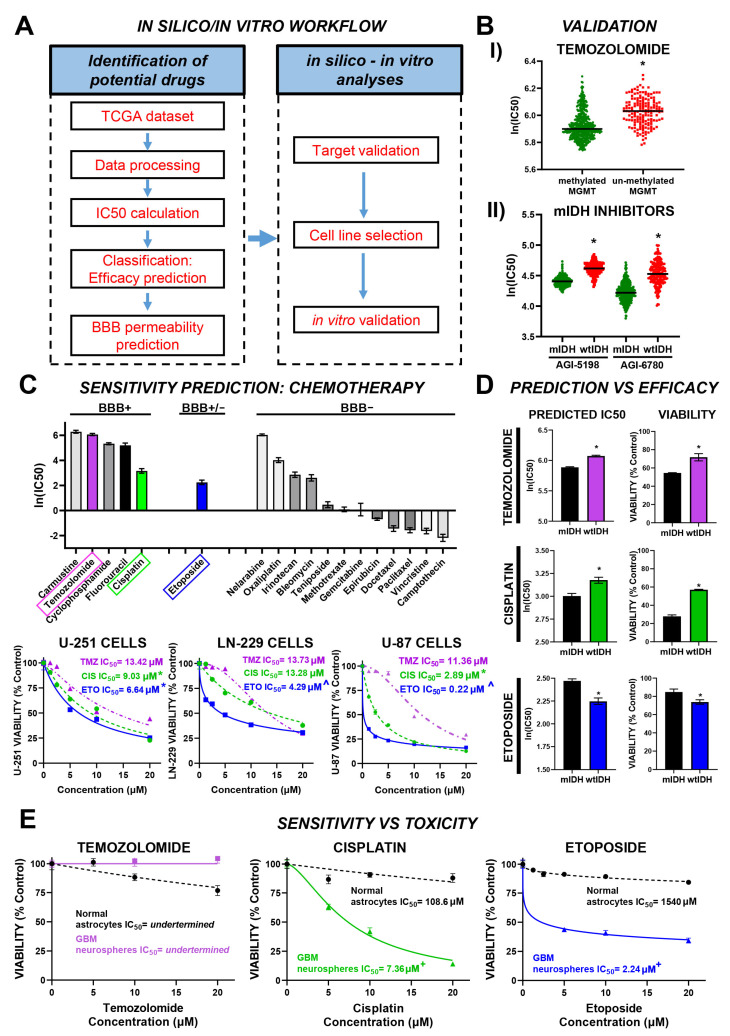
Efficacy prediction model for chemotherapeutic drugs with potential effect in GBM: (**A**) Schematic of the predictive pipeline used to identify alternative therapeutic candidates for glioblastoma. The process includes data processing of TCGA glioma datasets, IC50 estimation, efficacy prediction, and subsequent drug classification. Additional in silico assessments include blood-brain barrier (BBB) permeability predictions and target expression analysis, followed by selection of appropriate GBM cell lines for in vitro validation. (**B**) Validation of the algorithm using drugs with known or proposed activity in glioma. **I**) Predicted IC50 values for Temozolomide (TMZ) stratified by MGMT promoter methylation status (met: methylated; un-met: unmethylated). **II**) Predicted IC50 values for AGI-5198 and AGI-6780, selective inhibitors of mutant IDH1/2 (mIDH), in patients with either mIDH or wild-type IDH (wtIDH). All IC50 values are represented as ln(IC50). (**C**) Predicted IC50 values for conventional chemotherapeutics in wtIDH glioma patients compared to TMZ, categorized by BBB permeability prediction (BBB+: permeable; BBB+/−: ambiguous; BBB–: non-permeable). Commercial GBM cell lines (U251, LN229, U87) were treated with increasing concentrations of TMZ, Cisplatin, or Etoposide for 72 h. Cell viability was assessed by MTT assay in at least three independent biological replicates and six technical replicates per condition. Concentration–response curves were generated, and IC50 values were calculated using non-linear regression. * *p* < 0.05 vs. TMZ; ^ *p* < 0.05 vs. all other drugs (ANOVA). (**D**) Comparison of in silico predicted IC50 values and in vitro cytotoxicity for TMZ, Cisplatin, and Etoposide in mIDH and wtIDH glioma neurospheres. For in vitro validation, neurospheres were treated with fixed concentrations of each drug (TMZ: 15 µM; Cisplatin: 5 µM; Etoposide: 2.5 µM). Cell viability was measured by the MTT assay. (**E**) Cytotoxic effects of TMZ, Cisplatin, and Etoposide in normal murine astrocytes and GBM neurospheres. Cells were treated for 72 h with various drug concentrations, and IC50 values were determined as in C. * *p* < 0.05 vs. TMZ; ^ *p* < 0.05 vs. all other drugs (ANOVA); + *p* < 0.05 vs. normal astrocytes (Student’s *t*-test).

To identify chemotherapeutic agents with potential efficacy against glioblastoma (GBM), we employed a multi-step strategy integrating in silico drug sensitivity prediction with in vitro validation. Predicted IC50 values for 272 compounds, derived from TCGA transcriptomic data via the CancerRxTissue platform [18], were used to shortlist candidates. Our pipeline, summarized in Figure 1A, consists of three sequential stages: drug prioritization, computational screening, and experimental validation in GBM-relevant cellular models. In the initial stage, we stratified glioma patients according to the 2021 WHO CNS classification to estimate predicted IC50 values for each drug. This allowed us to identify compounds with the lowest median predicted IC50 across GBM samples, suggesting greater potential antitumoral activity. To identify potentially effective drugs, we applied the median of medians of predicted IC50 values as a selection threshold. The second stage included the following: (1) predicting blood-brain barrier (BBB) permeability; (2) assessing target expression in normal and glioma tissues to ensure tumor specificity; (3) prioritizing targets associated with poor prognosis in both progression-free interval (PFI) and overall survival (OS); and (4) selecting GBM cell lines with relevant expression levels of therapeutic targets. In the third stage, we experimentally validated the predictions using both normal and tumor-derived cell cultures.

To independently validate the predictive model developed by Li et al. [18], we first examined drugs commonly used in GBM with well-characterized clinical responses based on molecular features. As expected, the model predicts that temozolomide (TMZ) will have a stronger antitumor effect (lower IC50) in glioma patients with MGMT promoter methylation, which reduces expression of the DNA repair enzyme MGMT (Figure 1(BI)). Similarly, the model predicts greater sensitivity (lower IC50) to isocitrate dehydrogenase (IDH) inhibitors in patients with mutant IDH (mIDH) gliomas compared to wild-type IDH (wtIDH) cases (Figure 1(BII)). These results support the model’s potential for predicting drug responses in GBM.

Following the validation of the predictive model, we aimed to identify alternative chemotherapeutic agents for adult gliomas from a predefined drug panel (Figure 1C). To evaluate central nervous system bioavailability, we assessed blood-brain barrier (BBB) permeability using two independent in silico platforms, cBioligand [22] and ADMETlab3.0 [23]. Temozolomide (TMZ), carmustine, cyclophosphamide, fluorouracil, and cisplatin were consistently predicted as BBB permeable. Although predictions for Etoposide were inconclusive, its high in silico efficacy against GBM supported its inclusion in downstream analyses. Notably, carmustine and TMZ exhibited the highest predicted IC50 values, indicating lower antitumoral potency, whereas Etoposide and Cisplatin demonstrated superior predicted efficacy. Based on these findings, we selected Etoposide, Cisplatin, and TMZ—the standard-of-care agent—for further in silico and experimental validation. Concentration-response assays in U-251 MG, LN-229, and U-87MG glioma cell lines revealed greater sensitivity to Etoposide, followed by Cisplatin, compared to TMZ (Figure 1C), aligning with the computational predictions.

Comparative analysis of predicted IC50 values between mIDH and wtIDH gliomas revealed differential drug sensitivity profiles (Figure 1D). Specifically, wtIDH gliomas were predicted to be less responsive to temozolomide (TMZ) and cisplatin compared to mIDH gliomas, whereas Etoposide was predicted to be more effective in wtIDH tumors. These predictions were validated in vitro using neurospheres derived from genetically engineered murine models of mIDH and wtIDH gliomas [25], and treated with fixed concentrations of each drug (TMZ: 15 µM; Cisplatin: 5 µM; Etoposide: 2.5 µM) selected based on concentration–response assays in neurospheres to ensure comparable and submaximal cytotoxicity levels across drugs (Figure 1D).

**Figure 2:**
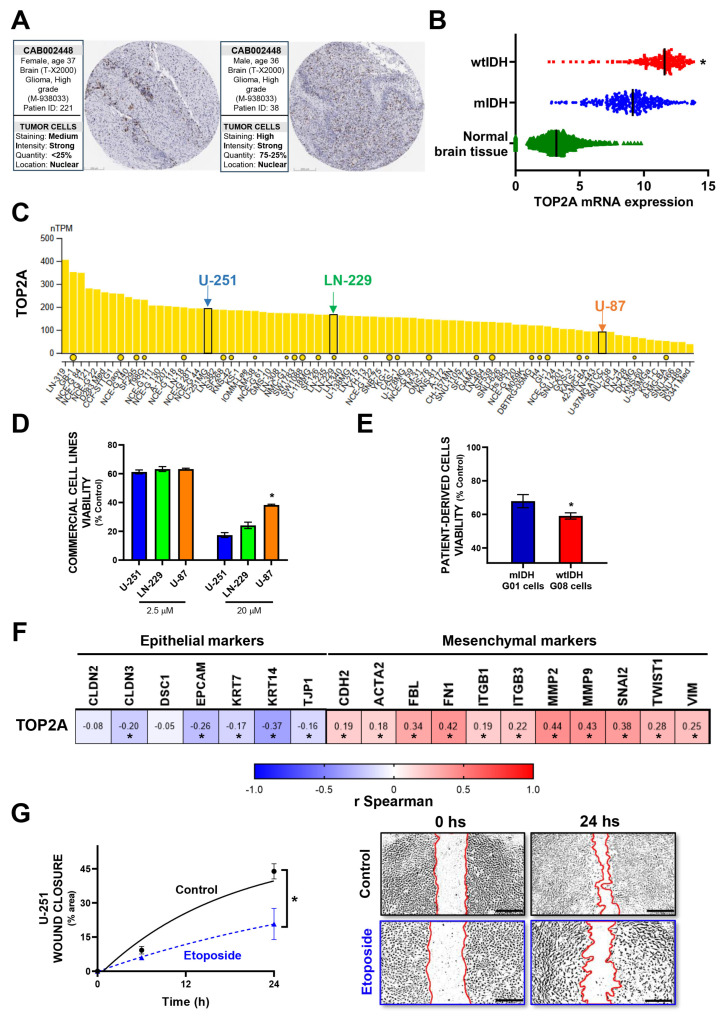
In Vitro Validation of Model-Predicted Etoposide Sensitivity in GBM Cells: (**A**) Representative immunohistochemistry (IHC) images of TOP2A expression in glioblastoma (GBM) patient biopsies obtained from the Human Protein Atlas (HPA). Scale bar, 200 µm. (**B**) Analysis of TOP2A mRNA expression in normal brain tissue versus glioma biopsies (mIDH and wtIDH), using datasets from TCGA LGG-GBM and GTEx. (**C**) TOP2A transcript levels in human GBM cell lines, based on HPA data. Expression values are shown as average transcripts per million (TPM) from replicate samples for each cell line. (**D**) Cytotoxic effects of Etoposide on U-251, LN229, and U-87 GBM cells with differential TOP2A expression. Cells were treated with increasing concentrations of Etoposide for 72 h, and viability was assessed via MTT assay. * *p* < 0.05 vs. U251 (ANOVA). (**E**) Patient-derived glioma cells with mIDH (G01) and wtIDH (G08) genotypes were treated with 2.5 µM Etoposide for 72 h, and cell viability was determined using the MTT assay. * *p* < 0.05 (Student’s *t*-test). (**F**) Spearman correlation between TOP2A expression and the expression of genes associated with the epithelial–mesenchymal transition (EMT). r: Spearman correlation coefficient. * *p* < 0.05. (**G**) U-251 GBM cells were grown to confluence and treated with 1 µM Etoposide for 24 h. Cell migration was assessed using a wound-healing assay. Wound area was quantified with ImageJ software, and closure was calculated relative to the area at time 0. Wound borders are outlined using red lines to aid visualization. Scale bar, 100 µm. * *p* < 0.05 vs. control (nonlinear regression analysis).

To evaluate potential off-target toxicity, we compared the cytotoxic effects of the selected drugs in primary cultures of normal murine astrocytes and GBM-derived neurospheres (Figure 1E). While TMZ induced a modest cytotoxic response in normal astrocytes, GBM neurospheres displayed limited sensitivity. Conversely, both Cisplatin and Etoposide demonstrated robust antitumoral activity in glioma cells with minimal cytotoxicity in normal astrocytes (Figure 2D). Based on its superior efficacy and favorable therapeutic index, Etoposide was selected for further evaluation.

### 3.2. In Vitro Validation of Model-Predicted Etoposide Sensitivity in GBM Cells

To explore the mechanistic basis of the model-predicted sensitivity to Etoposide, we first confirmed the expression of TOP2A, the gene encoding DNA topoisomerase II alpha—a known target of Etoposide [27]—in GBM tissues using data from The Human Protein Atlas (THPA) [24] (Figure 2A). In line with the model’s prediction of higher Etoposide sensitivity in wild-type IDH (wtIDH) gliomas compared to mutant IDH (mIDH) gliomas, transcriptomic analysis revealed significantly elevated TOP2A expression in wtIDH tumor samples relative to both mIDH gliomas and normal brain tissue (Figure 2B). We next examined TOP2A expression in commercial GBM cell lines. Based on THPA data [24], U-251 cells showed high expression, LN-229 intermediate, and U-87 low levels of TOP2A (Figure 2C). In vitro treatment with Etoposide revealed concentration-dependent cytotoxicity across all three lines, with higher concentrations (20 µM) eliciting responses that positively correlated with TOP2A expression levels (Figure 2D). These findings support a predictive relationship between TOP2A expression and Etoposide sensitivity in commercial glioma models.

To extend validation to patient-derived samples, we compared the response of G08 (wtIDH) and G01 (mIDH) glioma cultures to Etoposide [26]. Consistent with expression patterns observed in clinical samples (Figure 2B), G08 cells exhibited greater susceptibility to Etoposide-induced cytotoxicity than G01 cells (Figure 2E).

Transcriptomic profiling of GBM biopsies further revealed a positive correlation between TOP2A expression and epithelial–mesenchymal transition (EMT) markers (Figure 2F, Table S3). In line with these observations, TOP2 inhibition with Etoposide treatment significantly impaired GBM cell migration in wound-healing assays (Figure 2G), supporting its potential to inhibit both proliferative and invasive tumor behaviors.

### 3.3. Prediction Model for Alternative Drugs with Potential Therapeutic Effect in GBM

We next aimed to identify novel compounds, alternative to traditional chemotherapeutic drugs, with potential efficacy in GBM. We stratified glioma patients following the 2021 WHO CNS classification and analyzed predicted IC50 values, applying a median IC50 threshold to select candidates. Our multi-step workflow included assessing blood-brain barrier (BBB) permeability, evaluating target gene expression in glioma versus normal brain tissue, and selecting suitable GBM cell lines for subsequent in vitro validation.

**Figure 3:**
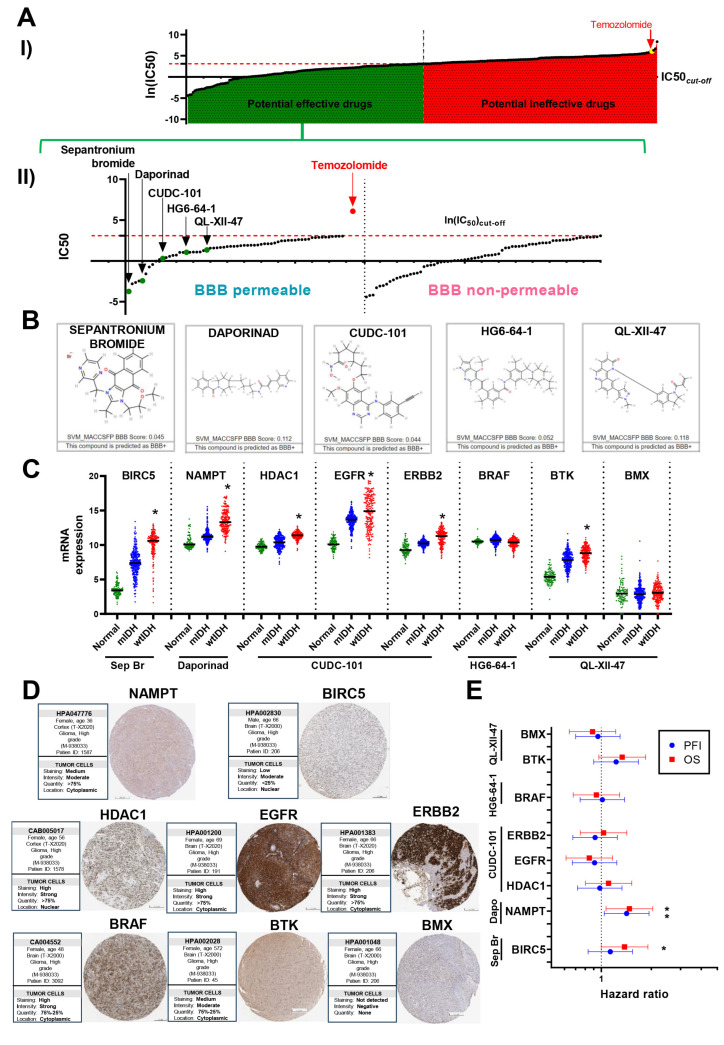
Prediction model for alternative drugs with potential therapeutic effect in GBM: (**A**) Identification of top candidate drugs for wtIDH glioma patients, based on efficacy predictions. (**I**) Drugs were classified as potentially effective or ineffective using the median of median predicted ln(IC50) values for 272 compounds as a cutoff. (**II**) Potentially effective drugs were further categorized based on predicted blood-brain barrier (BBB) permeability: BBB+ (permeable) and BBB− (non-permeable). A shortlist of five candidate drugs was generated according to efficacy and BBB+ status: Sepantronium bromide (BIRC5 inhibitor), Daporinad (NAMPT inhibitor), CUDC-101 (HDAC, EGFR, and ERBB2 inhibitor), HG6-64-1 (BRAF inhibitor), QL-XII-47 (BMX and BTK inhibitor). (**B**) Summary of BBB permeability predictions for the drugs evaluated in this study. (**C**) Analysis of mRNA expression levels of the molecular targets of selected drugs in normal brain tissue and glioma samples (mIDH and wtIDH), using transcriptomic data from TCGA. (**D**) Representative IHC images showing protein expression of the targets of shortlisted drugs in GBM patient biopsies, obtained from The Human Protein Atlas. Scale bar, 200 µm. (**E**) Forest plot analysis of progression-free interval (PFI) and overall survival (OS) in wtIDH glioma patients, stratified by high vs. low expression levels of each drug target. * *p* < 0.05, Log-rank (Mantel–Cox) test. All IC50 values are shown as natural log-transformed (ln IC50) values.

From 272 compounds in the CancerRxTissue dataset [18] (Figure 3(AI), Table S1), several BBB-permeable (BBB+) agents exhibited high predicted sensitivity against GBM, reflected by low IC50 values (Figure 3(AII), Table S2). Five candidates were shortlisted based on therapeutic relevance and BBB permeability: Sepantronium bromide (BIRC5 inhibitor), Daporinad (NAMPT inhibitor), CUDC-101 (HDAC, EGFR, HER2 inhibitor), HG6-64-1 (BRAF inhibitor), and QL-XII-47 (BMX and BTK inhibitor) (Figure 3B). Of note, all these compounds were predicted to be much more effective than TMZ, which exhibited one of the lowest therapeutic potentials amongst the predicted BBB+ drugs.

HG6-64-1 and QL-XII-47 were excluded due to low target gene upregulation in GBM (Figure 3C) and undetectable BMX expression in tumor samples (Figure 3D). Prognostic analyses of target genes using progression-free interval (PFI) and overall survival (OS) data further informed candidate prioritization (Figure 3E). Although Sepantronium bromide was predicted as BBB permeable, its rapid systemic clearance and limited brain accumulation [28,29] may reduce clinical utility in GBM. Moreover, BIRC5 expression correlated with OS but not with PFI. In contrast, NAMPT demonstrated consistent negative prognostic associations with both OS and PFI and was significantly overexpressed in GBM. Taken together, these data prioritized Daporinad (NAMPT inhibitor) for further preclinical evaluation (Figure 3D,E).

### 3.4. In Vitro Validation of Model-Predicted Daporinad Sensitivity in GBM Cells

**Figure 4:**
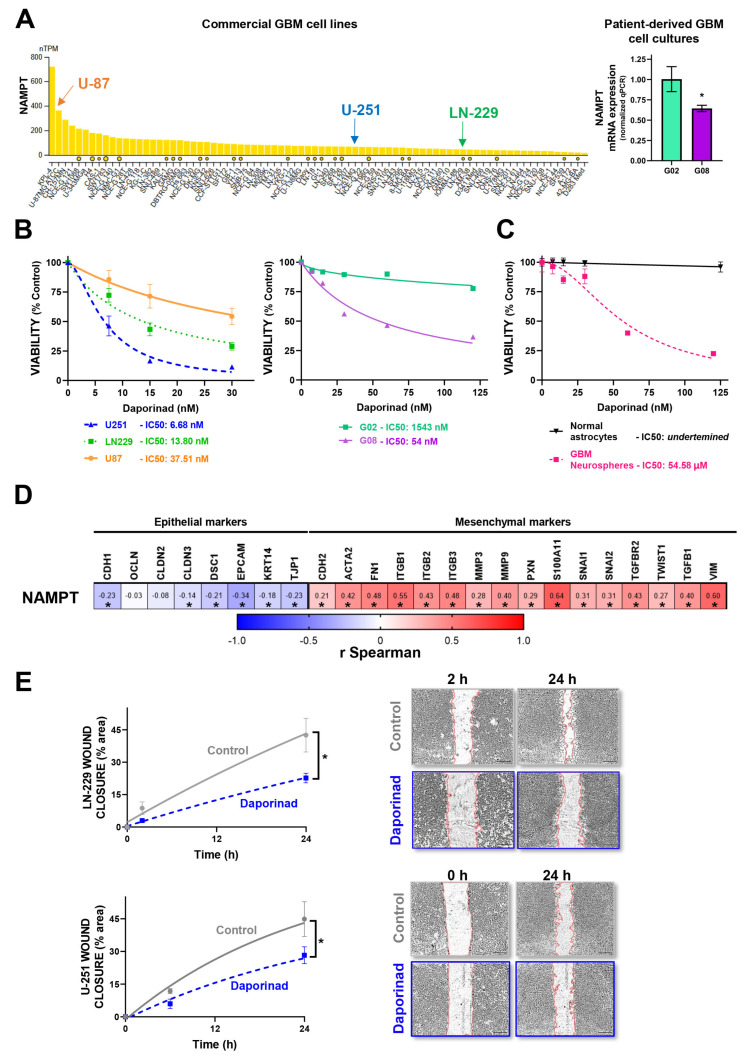
In Vitro Validation of Model-Predicted Daporinad Sensitivity in GBM Cells: (**A**) NAMPT mRNA expression levels in human GBM cell lines, using data from The Human Protein Atlas and relative NAMPT expression levels (RNA-seq) in patient-derived GBM cells G02 (NAMPThigh) and G08 (NAMPTlow). *, *p* < 0.05, Mann–Whitney test. (**B**) Evaluation of Daporinad cytotoxicity in commercial GBM cell lines (U-251, LN-229, U-87), patient-derived GBM biopsy-derived cultures, and (**C**) murine normal astrocytes and GBM neurospheres, following treatment with increasing concentrations of Daporinad for 72 h. Cell viability was assessed by MTT assay, and IC50 values were calculated using nonlinear regression from concentration–response curves. (**D**) Spearman correlation analysis between NAMPT expression and proteins involved in epithelial–mesenchymal transition (EMT). r, Spearman coefficient. * *p* < 0.05. (**E**) Evaluation of cell migration in LN-229 and U-251 cells. After reaching confluence, cells were treated with Daporinad (10 nM) for 24 h, and wound closure assays were performed at multiple time points. Wound area was quantified with ImageJ software, and closure was calculated relative to the area at time 0. Wound borders are outlined using red lines to aid visualization. * *p* < 0.05 vs. control (nonlinear regression analysis).

We evaluated NAMPT mRNA expression in commercial GBM cell lines and patient-derived GBM cultures using transcriptomic data from The Human Protein Atlas (THPA) (Figure 4A), selecting models with varying NAMPT levels for functional validation. In vitro cytotoxicity assays demonstrated an inverse correlation between NAMPT expression and sensitivity to Daporinad: U-87 cells, with the highest NAMPT expression, exhibited the lowest sensitivity, whereas U-251 cells, expressing the least NAMPT, were most sensitive. LN-229 cells, with intermediate NAMPT expression, showed moderate sensitivity (Figure 4B, left). This pattern was mirrored in patient-derived GBM cultures, where G02 cells (low NAMPT) were more sensitive than G08 cells (high NAMPT) (Figure 4B, right). Despite this inverse correlation, Daporinad induced potent cytotoxic effects across all human GBM models tested (U-87, U-251, LN-229, G02, and G08) (Figure 4B), as well as in murine GBM neurospheres (Figure 4C), while sparing normal murine astrocytes (Figure 4C).

Further transcriptomic analysis of TCGA GBM samples revealed a positive correlation between NAMPT expression and epithelial-to-mesenchymal transition (EMT) markers (Figure 4D, Table S3), suggesting a link between NAMPT and a mesenchymal tumor phenotype. Consistent with this association, NAMPT inhibition with Daporinad significantly inhibited GBM cell migration in wound-healing assays (Figure 4E), supporting its potential to limit tumor invasiveness.

### 3.5. Identification of Drug Combinations with Potential Therapeutic Efficacy in GBM

**Figure 5:**
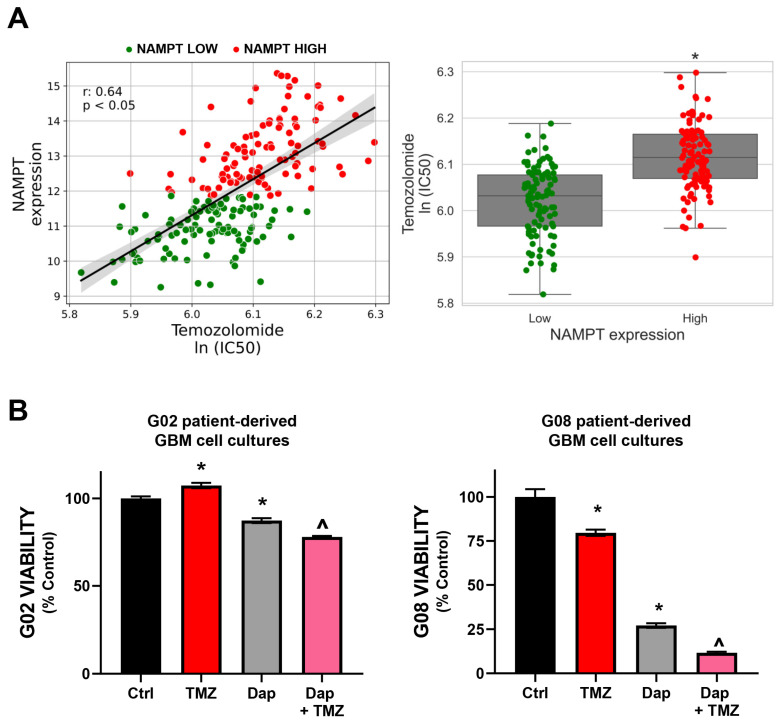
Potential of Daporinad in combination with Temozolomide (TMZ) in GBM cells: (**A**) Spearman correlation between NAMPT expression and TMZ predicted efficacy (left panel) in GBM patients. Green and red dots represent NAMPT low and high expression levels, respectively. Predicted ln(IC50) values for TMZ in wtIDH glioma patients, stratified by NAMPT expression (right panel). Patients were classified into “Low” and “High” NAMPT expression groups using the median expression value as a threshold. *p* < 0.05; Mann–Whitney test. (**B**) Cell viability of G02 and G08 cells treated with Daporinad (60 nM), Temozolomide (150 µM), or their combination for 72 h, assessed by MTT assay. *, *p* < 0.05 vs. control; ^, *p* < 0.05 vs. drugs alone. ANOVA with Tukey’s multiple comparisons test.

To identify potential combinations for GBM, we examined the correlation between NAMPT mRNA expression and drug response in patient-derived tumor biopsies from TCGA. A positive correlation between TMZ ln(IC50) and NAMPT expression was observed, indicating that higher NAMPT levels may decrease TMZ sensitivity (Figure 5A). This suggests that combining Daporinad with TMZ could enhance chemosensitivity, particularly in tumors with elevated NAMPT. To validate this, we used patient-derived GBM cultures with distinct NAMPT expression profiles determined by RNA-seq: NAMPT_high_ (G02) and NAMPT_low_ (G08) (Figure 4A). Cells were exposed to Daporinad (60 nM), TMZ (150 μM), or their combination. Consistent with NAMPT expression, G08 cells (NAMPT_low_) showed greater sensitivity to both drugs, while G02 cells (NAMPT_high_) were resistant to TMZ and less sensitive to Daporinad (Figure 5B). Notably, the combination treatment improved sensitivity in both cell lines, with a more pronounced effect in G08 cells. These findings align with our predictive model, reflecting reduced responsiveness in NAMPT_high_ cells.

Subsequently, we integrated Daporinad predicted sensitivity with gene expression data from canonical signaling pathways (Figures S1 and S2, Table S4). TCGA GBM patients were stratified into “high” and “low” expression groups for key pathways [30], and Daporinad IC50 values were compared accordingly. Elevated IC50 values in the high-expression groups implied that co-targeting these pathways may potentiate Daporinad’s efficacy. This analysis revealed promising combination strategies involving Daporinad and inhibitors of HDAC, HER2, PI3K, VEGF, ALK, CTLA4, RAS, RB, retinol metabolism, and estrogen signaling pathways. These results provide a rationale for multi-targeted combination therapies aiming to improve clinical outcomes by overcoming resistance mechanisms [31,32,33].

## 4. Discussion

In the era of personalized medicine, glioblastoma (GBM) continues to present formidable challenges. Efforts to develop genomic-driven therapies have been largely unsuccessful, partly due to the reliance on targeting individual tumor features with single-agent treatments. This strategy is delayed by the profound genomic heterogeneity of GBM, which involves spatial, temporal, inter-, and intra-tumoral variability [34,35]. While some novel therapies have demonstrated encouraging effects in preclinical models [36], the lack of clinical success underscores the need for alternative approaches. One such strategy is drug repurposing, which leverages well-established drugs that are often safer, more affordable, and more readily translatable from bench to bedside.

Li et al. [18] developed predictive models for 272 drugs by integrating gene expression data with drug sensitivity (IC50) profiles from cancer cell lines. These models were then applied to RNA-seq data from The Cancer Genome Atlas (TCGA) to estimate drug sensitivity in tumor tissues. If validated, this approach could significantly influence preclinical drug testing and early-phase clinical trial design. We developed a novel workflow to prioritize candidate drugs for GBM by not only considering predicted efficacy, but also by filtering out compounds with low target relevance or inadequate brain penetration. For example, although the blood-brain barrier (BBB) permeability of Etoposide remains uncertain, it has been used in GBM patients and may transiently disrupt the BBB to improve central nervous system (CNS) access [37,38,39]. Its favorable in vitro therapeutic index and low expression of its target, TOP2A, in normal brain tissue further support its potential for GBM treatment [40,41,42,43,44]. Other agents, such as Irinotecan, Methotrexate, Vincristine, and Paclitaxel—despite limited BBB penetration—were predicted to outperform standard drugs like Temozolomide, Cisplatin, and Etoposide. Thus, enhancing their distribution within glioma tissues [45] could further increase their therapeutic efficacy and broaden their clinical utility in GBM.

Using our innovative workflow, we identified numerous drugs with therapeutic potential for GBM, including non-traditional compounds, and established a shortlist of five candidates with different mechanisms of action: Sepantronium bromide, Daporinad, CUDC-101, HG6-64-1, and QL-XII-47. While our approach builds upon the IC50 predictions from Li et al. [18], it differs significantly in its objectives and methodology. Li et al. focused on generating large-scale, in silico drug sensitivity profiles across cancer types using gene expression data from cell lines and subsequently extrapolated this to tumor and normal tissues from TCGA and GTEx. In contrast, our goal was to refine and prioritize candidates specifically curated for glioblastoma treatment, integrating context-specific parameters such as blood-brain barrier (BBB) permeability, differential target expression in tumor versus normal brain, and prognostic value of drug targets.

This multi-layered filtering pipeline improves biological and clinical relevance, even if it is more selective in scope. Importantly, our strategy is also time-efficient in translational terms, as it narrows the focus to compounds with a higher likelihood of success in GBM-specific preclinical and clinical settings. For instance, unlike Li et al., who highlighted Bcl-2 family inhibitors such as ABT-737, navitoclax, and venetoclax, we prioritized agents with mechanisms involving mitotic arrest, NAD biosynthesis inhibition, or epigenetic modulation. These selections were further validated experimentally using in vitro models, underscoring our emphasis on functional relevance and feasibility. While a limitation of our workflow is that it does not independently generate IC50 predictions, it leverages existing computational models and complements them with stringent, disease-relevant criteria to accelerate candidate prioritization for translational research.

We placed particular emphasis on Daporinad, a NAMPT inhibitor with previously reported antitumor activity and favorable safety profiles in early-phase clinical trials for refractory B-cell chronic lymphocytic leukemia and advanced melanoma [46,47,48,49]. NAMPT is the rate-limiting enzyme in the biosynthesis of nicotinamide adenine dinucleotide (NAD+), a metabolite essential for numerous cellular processes in both normal and malignant cells [50,51]. This dependence is especially pronounced in glioblastoma, where tumor cells exhibit heightened NAD^+^ demand [52].

Emerging evidence underscores the importance of NAMPT in GBM biology. Its overexpression is associated with glioma stem cell maintenance and radioresistance [53,54,55]. Although first-generation NAMPT inhibitors have demonstrated limited efficacy and significant toxicity in GBM [56], genetic knockdown of NAMPT in GBM cells has been shown to reduce proliferation, migration, and invasion, while promoting apoptosis [53]. Moreover, NAMPT inhibitors can sensitize GBM cells to temozolomide (TMZ) by activating the ROS/JNK signaling pathway [57]. Recent advances in targeted drug delivery and the development of NAMPT degraders offer promising strategies to improve the therapeutic utility of NAMPT inhibition in GBM [58].

Despite encouraging preclinical findings, Daporinad has not consistently demonstrated antitumor efficacy in clinical trials across other cancer types [48,49,59]. Efforts to improve NAMPT inhibitors include identifying predictive biomarkers and developing compounds with enhanced pharmacodynamics [58]. Emerging evidence suggests that the gut microbiota may influence NAMPT inhibitor efficacy, with some microorganisms potentially reducing the cytotoxicity of Daporinad by increasing NAD+ biosynthesis [60,61]. While preliminary, this points to microbiota modulation—such as via antibiotics—as a possible strategy to enhance treatment, though further research is needed to confirm these effects [60,61].

Our results suggest that Daporinad-mediated NAMPT inhibition may enhance TMZ sensitivity in GBM, consistent with a recent report demonstrating synergistic effects in both GBM cell lines in vitro [57] and animal models in vivo [62]. The drug predictions revealed by our workflow are consistent with independent findings, supporting its utility as a predictive tool for identifying effective drug candidates and rational combination strategies with the potential to improve therapeutic outcomes in GBM. However, the absence of formal synergy analyses, such as Combination Index or Bliss independence models, represents a limitation of the current study. Future investigations should apply rigorous synergy quantification methods to quantitatively assess these interactions and further validate their therapeutic potential in vivo.

Furthermore, our approach identifies prospective drug combinations involving Daporinad and agents targeting complementary pathways, providing a hypothesis-generating framework for preclinical evaluation. By bridging preclinical data with clinical relevance, this strategy promotes robust and translationally meaningful findings. The use of patient-derived GBM cells enhances the physiological relevance of our model, facilitating more accurate predictions of clinical efficacy. Importantly, our in vitro toxicity assays—performed on both GBM cells and normal astrocytes—ensure that selected compounds possess therapeutic selectivity, reducing the likelihood of adverse effects on healthy brain tissue and supporting the identification of drugs with favorable therapeutic indices.

Given that TCGA comprises over 10,000 samples across 32 cancer types, our workflow offers a flexible and scalable platform for therapeutic prioritization. By screening a library of 272 compounds, most of which are already approved for other clinical indications, this approach enables broad applicability and individualized treatment selection based on the molecular profiles of patient tumors. Furthermore, by aligning drug predictions with patient-specific genomic data, our model holds potential for integration into clinical workflows to support real-time decision making in personalized oncology, for example, using sequencing data to predict drug sensitivity and guide treatment selection tailored to each patient.

While our workflow shows promise, it also has its limitations. First, in vivo validation is still needed to confirm the efficacy and safety of prioritized drugs in a more realistic tumor model. Second, predictions of blood-brain barrier (BBB) permeability rely on in silico models, which may not fully reflect pharmacokinetics in patients and therefore require experimental confirmation. Third, our analysis is based on transcriptomic data to assess drug target expression, which may not always correlate with protein levels or functional activity. Incorporating proteomic profiling and functional assays will help address this limitation. Nevertheless, transcriptomic data from the biopsies are more often available in the clinic than proteomic data. Future directions include in vivo evaluation of selected compounds, formal synergy testing for promising drug combinations (e.g., with TMZ), and development of targeted delivery strategies to improve BBB penetration and therapeutic efficacy. Addressing these aspects will be essential to strengthen the clinical relevance and translational potential of this approach.

## 5. Conclusions

Our workflow successfully identified and validated alternative drug candidates and therapeutic combinations for GBM, demonstrating efficacy and safety in preclinical models, including patient-derived cells and normal astrocytes. This approach introduces novel pharmacological strategies for a disease that has seen limited therapeutic advancement over the past two decades. By prioritizing the repurposing of non-toxic drugs already tested clinically for other indications, our approach leverages existing safety data to potentially accelerate clinical translation. Overall, this platform may help optimize resource utilization, reduce reliance on animal testing, and facilitate the identification of effective treatment combinations for GBM, representing a potentially valuable tool to support therapeutic development.

## Data Availability

The data presented in this study were derived from the following resources available in the public domain: Xena Browser: https://xenabrowser.net/. CanceRxTissue: https://manticore.niehs.nih.gov/cancerRxTissue/.

## Supplementary Materials

The following supporting information can be downloaded at: https://www.mdpi.com/article/10.3390/brainsci15060637/s1. Figure S1. Predictive effect of Daporinad according to the expression of targets for alternative drugs. Daporinad predicted IC50 for GBM patients classified according to canonical pathways expression levels into “Low” (green) and “High” (red) expression, using the median of expression of each pathway as a cut-off. * *p* <0.05; Mann–Whitney test. Figure S2. Correlations of Daporinad’s predictive effect with the expression of targets for alternative drugs. Spearman correlation between Daporinad predicted IC50 for GBM patients and expression levels of specific canonical pathways. Each point represents a sample, color coded by the expression level of the pathway (green: Low, red: High). Black lines indicate linear regression. r, Spearman coefficient. Table S1. Predicted Drug Sensitivity Rankings in GBM from the CancerRxTissue Dataset. Table S2. Predicted BBB Permeability and Sensitivity of Candidate Drugs in GBM. Table S3. Pearson correlation of TOP2A and NAMPT expression with epithelial and mesenchymal gene markers in GBM. Table S4. Pathway-Integrated Analysis of Daporinad Sensitivity and Potential Combinatorial Targets.
