# Supplementary figures and images for: Integrated Workflow for Drug Repurposing in Glioblastoma: Computational Prediction and Preclinical Validation of Therapeutic Candidates
Source: Brain Sci. 2025 Jun 13;15(6):637. doi: 10.3390/brainsci15060637 (PMC12191016; doi:10.3390/brainsci15060637)

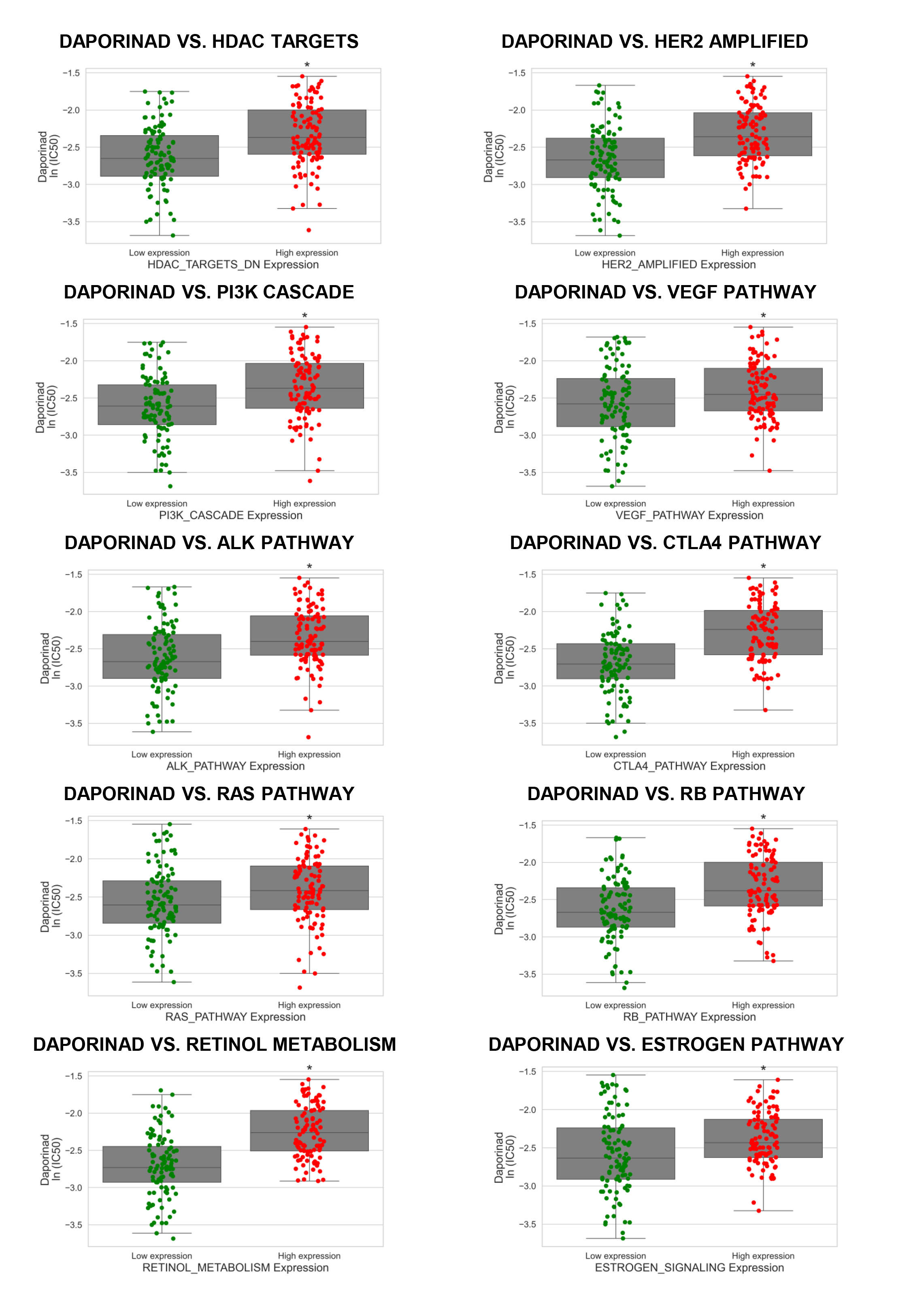

Supplement: Supplementary file 1 [file brainsci-15-00637-s001.zip › FIGURE S1.tif]

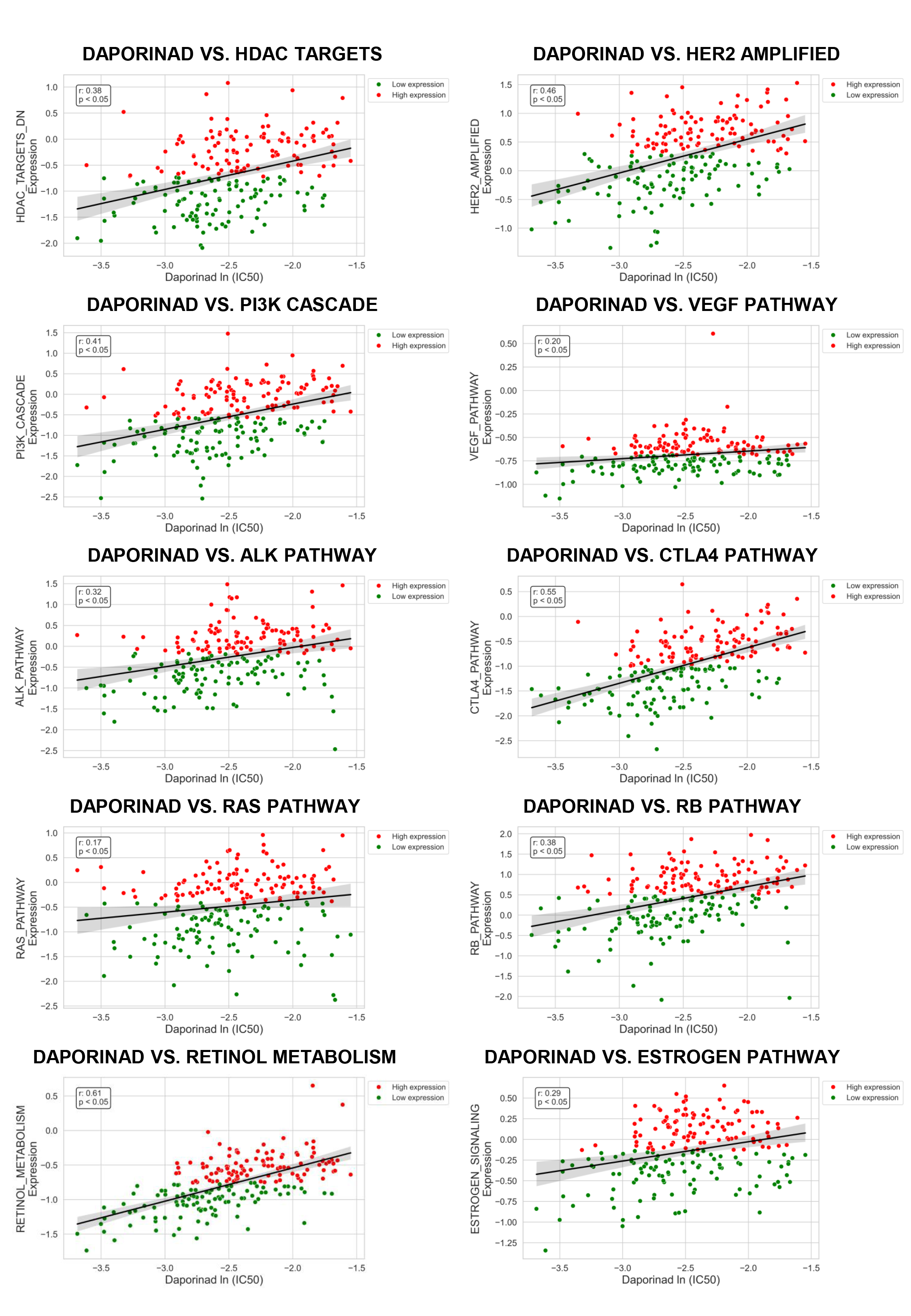

Supplement: Supplementary file 1 [file brainsci-15-00637-s001.zip › FIGURE S2 (1).tif]
